# Quantum Attack-Resistent Certificateless Multi-Receiver Signcryption Scheme

**DOI:** 10.1371/journal.pone.0049141

**Published:** 2013-06-05

**Authors:** Huixian Li, Xubao Chen, Liaojun Pang, Weisong Shi

**Affiliations:** 1 School of Computer Science and Engineering, Northwestern Polytechnical University, Xi’an, China; 2 Department of Computer Science, Wayne State University, Detroit, Michigan, United States of America; 3 School of Life Sciences and Technology, Xidian University, Xi’an, China; University of Nottingham, United Kingdom

## Abstract

The existing certificateless signcryption schemes were designed mainly based on the traditional public key cryptography, in which the security relies on the hard problems, such as factor decomposition and discrete logarithm. However, these problems will be easily solved by the quantum computing. So the existing certificateless signcryption schemes are vulnerable to the quantum attack. Multivariate public key cryptography (MPKC), which can resist the quantum attack, is one of the alternative solutions to guarantee the security of communications in the post-quantum age. Motivated by these concerns, we proposed a new construction of the certificateless multi-receiver signcryption scheme (CLMSC) based on MPKC. The new scheme inherits the security of MPKC, which can withstand the quantum attack. Multivariate quadratic polynomial operations, which have lower computation complexity than bilinear pairing operations, are employed in signcrypting a message for a certain number of receivers in our scheme. Security analysis shows that our scheme is a secure MPKC-based scheme. We proved its security under the hardness of the Multivariate Quadratic (MQ) problem and its unforgeability under the Isomorphism of Polynomials (IP) assumption in the random oracle model. The analysis results show that our scheme also has the security properties of non-repudiation, perfect forward secrecy, perfect backward secrecy and public verifiability. Compared with the existing schemes in terms of computation complexity and ciphertext length, our scheme is more efficient, which makes it suitable for terminals with low computation capacity like smart cards.

## Introduction

Signcryption is a cryptographic primitive that provides both signature and encryption simultaneously to sensitive information at a lower computation and communication overhead than the traditional signature-then-encryption approach [1]. In terms of the implementation method, there are two kinds of signcryption schemes. One is based on traditional public key infrastructure [2], which causes the costly certificate management problem; the other is based on identity-based public key cryptography [3], which avoids the certificate management, but induces the key escrow problem.

In 2003, Al-Riyami et al. [4] proposed the certificateless cryptosystem (CLC). In their certificateless cryptosystem, a user’s secret key is derived from two parts: one is an identity-based secret key generated by Key Generation Center (KGC) and the other is a self-generated secret key. Thus CLC solves the key escrow problem as well as the certificate management problem, and it also reduces the implementation complexity of the cryptosystem. In 2008, Barbosa et al. [5] first proposed the certificateless signcryption scheme (CLSC) based on bilinear pairing operations. However, they did not give the security proof of their scheme. Since then, certificateless signcryption schemes [6]–[7] have been studied extensively. In 2010, Li et al. proposed another CLSC [8] and proved its security formally. However, these schemes [5]–[8] are inefficient in computation because they use the bilinear pairing operation, a quite complex computation. Selvi et al. [9] and Jing et al. [10] constructed an efficient CLSC based on the CDH (Computational Diffie-Hellman) problem without bilinear pairing operations, respectively. At the same time, they proved their schemes’ security in the random oracle model. However, their schemes are only single-receiver ones. If there are multiple receivers at the same time, these schemes need to signcrypt the same message for each receiver separately, so they are very inefficient for the multi-receiver scenario. In order to improve the efficiency of signcryption in the multi-receiver setting, Selvi et al. [11] proposed the certificateless multi-receiver signcryption scheme (CLMSC), and this scheme only needs two bilinear pairing operations and (*t*+2) exponentiation operations (*t* denotes the number of the receivers) in the signcryption and designcryption phases. However, they found that this scheme cannot resist the forgery attack and then presented an enhanced scheme [12] later. But Miao et al. [13] showed that the enhanced scheme is still insecure against the internal attack and provided a detailed security analysis.

To date, the implementations of almost all certificateless signcryption schemes [5]–[13] are based on traditional public key cryptosystems, in which the security mainly relies on the hard problems, such as factor decomposition and discrete logarithm. However, in 1994, Shor [14] proposed a polynomial-time quantum algorithm that can successfully factor large integers, which shows that quantum computing has brought a potential challenge to these hard mathematical problems. Once quantum computers is developed successfully, they will pose a fatal threat to the security of almost all certificateless signcryption schemes which are based on public key cryptosystems such as RSA, ElGamal and ECC. So it is more and more urgent to design a certificateless signcryption scheme that can resist quantum attack. Multivariate public key cryptography (MPKC), which can resist quantum attack, is one of the alternative solutions to guarantee the security of communications in the post-quantum age. The security of MPKC is based on the Multivariate Quadratic (MQ) problem and the Isomorphism of Polynomials (IP) problem. Compared with identity-based cryptography, MPKC has lower calculation complexity and is higher in efficiency, which makes MPKC well suitable to implement strongly secure communications for low-end devices. The MPKC-based schemes have been studied widely, and several excellent schemes have been proposed. For example, SFLASH, a signature scheme based on MPKC, has been recommended by the NESSIE European Consortium since 2003 as the best known solution for implementation on low-cost smart cards [15].

### 

#### Our contribution

Motivated by these concerns, we employ MPKC to construct an efficient quantum attack-resistent certificateless multi-receiver signcryption scheme, which combines the certificateless cryptosystem and MPKC. The new scheme not only has the advantage of the certificateless cryptosystem, which avoids the problem of key management, but also resists quantum attack only with light-weight computation like the multivariate quadratic polynomial operations. In our scheme, multivariate quadratic polynomial operations, which have lower computation complexity than bilinear pairing operations, are employed in signcrypting a message for a certain number of receivers. Therefore, our scheme is more efficient than the existing CLMSC schemes, and it is suitable for mobile terminals with low computing power. Security analysis shows that our scheme is a secure MPKC-based multi-receiver signcryption scheme, and it also has the important security properties such as message confidentiality, unforgeability, non-repudiation, perfect forward secrecy, perfect backward secrecy and public verifiability.

## Preliminaries

### 1 MQ Problem and IP Problem

In this section, we shall briefly recall some basic concepts of MPKC including multivariate polynomial equations, the MQ problem and the IP problem.

Let *G* be a finite field of prime order *p*. Let *n* be the number of variables, namely, *x*_1_, *x*_2_, …, *x_n_* in the multivariate polynomial equation, *g* be the number of the multivariate polynomial equations, and *d* be the degree of the multivariate polynomial equations.

A tuple of multivariate quadratic polynomials consists of a finite ordered set of polynomials of the following form:
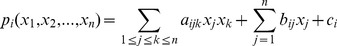
(1)where *i = *1, 2, …, *g*, and *x_j_*, *x_k_*∈*G*, and the coefficients {*a_ijk_*, *b_ij_*, *c_i_*} are over *G* [16]. Then, the MQ problem can be described as follows:

#### Definition 1

(MQ). Given a tuple *P* =  (*p*_1_, *p*_2_, …, *p_g_*) of *g* multivariate quadratic polynomials with *n* unknowns defined over *G*, and the image *y* = (*p*_1_(*z*), *p*_2_(*z*), …, *p_g_*(*z*)) of an element *z* randomly chosen from *G^n^*(*G^n^* denotes the *n*th extension of *G*), the problem to find an element *x* of *G^n^* such that *y* = (*p*_1_(*x*), *p*_2_(*x*), …, *p_g_*(*x*)) is called the MQ problem [16].

Solving a set of randomly chosen quadratic equations with several variables over a finite field is considered as an NP hard problem [17].

#### Definition 2

(IP). Given *P* and *Q* be two public sets of *n* quadratic equations with *n* variables over *G*, if *P* and *Q* are isomorphism, then 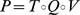
(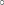 denotes composition of mappings), where *T* and *V* are two invertible affine transformations on 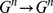. Finding (*T*, *V*) for *P*, *Q* such that 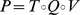 is called the IP problem [18].

### 2 Multivariate Public Key Cryptosystem

In a primitive multivariate public key cryptosystem [19], for a user *U* with identity ID*_U_*, his/her public key is 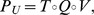 and his/her secret key is the 3-tuple 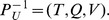 The encryption operation for a message *m* is denoted by *σ = P_U_*(*m*) and the corresponding decryption operation for the ciphertext *σ* is denoted by 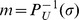. For example, Alice wants to send a message *m* to Bob with identity ID*_B_*. Alice computes the ciphertext 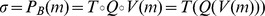
with Bob’s public key. Bob receives the ciphertext *σ* from Alice and then decrypts the ciphertext *σ* by computing 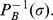 In a word, Bob computes 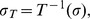 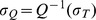
and 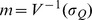 sequentially. At last, Bob obtains the plaintext message 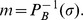


### 3 Framework of CLMSC

A certificateless multi-receiver signcryption scheme consists of five probabilistic polynomial-time algorithms, namely Setup, Partial Key Extract, Key Extract, Signcrypt and De-signcrypt. According to the features of MPKC, we improve the existing CLMSC model, that is, KGC produces the common partial public key and partial secret key in the phase of Partial Key Extract.

- **Setup**: This algorithm is run by KGC. It takes as input a security parameter *s* and returns the public parameters *params*.
- **Partial Key Extract**: This algorithm is run by KGC. KGC first chooses a random number *w* as the system master key. Then it takes as input *w* and *params* and returns the common partial public key *PP_u_* and partial secret key *PS_u_*.
- **Key Extract**: This algorithm is run by a user *U*. It takes as input *params*, *PP_u_*, *PS_u_* and an identity ID*_u_* and returns the full public key *PK_u_* and the full secret key *SK_u_* of the user.
- **Signcrypt**: To securely send a message *m* to a group of receivers {ID_1_, ID_2_, …, ID*_t_*}, the sender *S* should run this algorithm to signcrypt it first. It takes as input *params*, a message *m*, the sender’s identity ID*_S_*, the full keys *PK_u_* and *SK_u_* of the sender, and lists of the receiver identities and their public keys, and returns a ciphertext *σ*.
- **De-signcrypt**: This algorithm takes as input a ciphertext *σ*, the receiver’s identity ID*_i_*, the receiver’s full keys *PK_i_* and *SK_i_*, the identity ID*_s_* and the public key *PK_s_* of the sender, and returns either a plaintext *m* or an error symbol ⊥.

### 4 Security Model for CLMSC

Our security model is established based on Selvi et al.’s security model [11]. For a certificateless signcryption scheme, there are two types of attacks corresponding to two types of attackers, namely *A*_1_ and *A*_2_. In the attack of Type 1, *A*_1_ does not have access to the system master key, but he/she has the ability to replace the public key of any user with a value that he/she chooses arbitrarily. *A*_2_ has access to the master key, but he/she cannot change public key of any user.

#### Definition 3

Confidentiality under the attack of Type 1. A certificateless multi-receiver signcryption scheme is Type-1-CCA2 secure if no probabilistic polynomial-time attacker *A* has a non-negligible advantage in winning the IND-CLMSC-CCA2-1 game [11].

For *A*, there are the following constraints. *A* can not have access to the master key *w*. No Extract Secret Key query is allowed on any of the challenge identities. No De-signcrypt query is allowed on the challenge ciphertext.

#### Definition 4

Confidentiality under the attack of Type 2. A certificateless multi-receiver signcryption scheme is Type-2-CCA2 secure if no probabilistic polynomial-time attacker *A* has a non-negligible advantage in winning the IND-CLMSC-CCA2-2 game [11].

For *A*, there are the following constraints. No Extract Secret Key query is allowed on any of the challenge identities. No Replace Public Key query is allowed on any of the challenge identities. No De-signcrypt query is allowed on the challenge ciphertext.

#### Definition 5

Unforgeability under the attack of Type 1. A certificateless multi-receiver signcryption scheme is Type-1-sEUF-CMA-1 secure if no probabilistic polynomial-time attacker *A* has a non-negligible advantage in winning the EUF-CLMSC-CMA-1 game [11].

For *A*, there are the following constraints. *A* can not have access to the master key *w*. No Extract Secret Key query is allowed on any of the challenge identities.

#### Definition 6

Confidentiality under the attack of Type 2. A certificateless multi-receiver signcryption scheme is Type-2-sEUF-CMA-2 secure if no probabilistic polynomial-time attacker *A* has a non-negligible advantage in winning the EUF-CLMSC-CMA-2 game [11].

For *A*, there are the following constraints. No Extract Secret Key query is allowed on any of the challenge identities. No Replace Public Key query is allowed on any of the challenge identities.

## Methods

In order to construct our scheme, we employed the Perturbed Matsumoto-Imai-Plus (PMI+) cryptosystem [20], which can resist the linearization attack, rank attacks, and the differential attack and is much faster than RSA and ECC. The new scheme that we proposed consists of five probabilistic polynomial-time algorithms, namely Setup, Partial Key Extract, Key Extract, Signcrypt and De-signcrypt. We shall give a detailed description of the proposed scheme as follows.

### 

#### Setup

Given a security parameter *s* as input, KGC returns a big positive integer *q* and a small positive integer *p.* Let *G* be a finite field of order *q* and characteristic two, and define two non-collision hash functions *H*_1_: 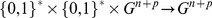
and *H*_2_: 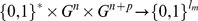, where *G^n^* is the *n*th extension of *G* and *G^n^* ^+*p*^ is the (*n*+*p*)th extension of *G*. The positive integer *n* is the number of variables in the equation (1) and *l_m_* is the bit length of the message *m*. Then KGC selects a positive integer *g* to denote the number of equations. At last, KGC publishes the public parameters *params* denoted by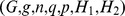.

### Partial Key Extract

1. KGC selects a secure MPKC, which is PMI+ in our scheme. The system public key can be expressed as a typical multivariate quadratic system: 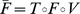 where *T* is a randomly chosen invertible affine transformation on 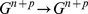, *V* is a randomly chosen invertible affine transformation on 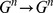, and *F* is a public set of (*n*+*p*) quadratic equations with *n* variables over *G*. The system secret key is (*T*, *F*, *V*). The related parameters refer to [20].
2. KGC randomly chooses *T*_0_ and *V*_0_, where *T*_0_ is a randomly chosen invertible affine transformation on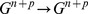 and *V*_0_ is a randomly chosen invertible affine transformation on 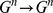. Then, compute 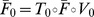. 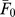 is the system partial public key, and 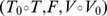 is the system partial secret key. It is worth noting that KGC needs to compute the new system partial secret key when some user drops out of the group.

#### Key Extract

Each user runs this algorithm to compute his/her full public and secret keys. The user *U* randomly chooses *T_u_* and *V_u_*, where *T_u_* is an invertible affine transformation on 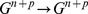
and *V_u_* is an invertible affine transformation on 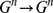. Then, compute the public key *F_u_* of user *U*, that is, 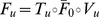, which should be sent to KGC. The secret key of user *U* is (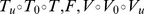).

#### Signcrypt

Suppose that Alice, whose identity is ID*_A_*, wants to signcrypt a message *m* to *t* different receivers denoted by *L* =  {ID_1_, ID_2_, …, ID*_t_*}. Alice performs the following steps:

1. Alice chooses 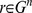 randomly, and computes 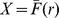,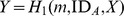 and 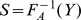.
2. For all IDi, i = 1, 2, …, t, compute 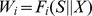
and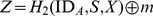.
3. Return ciphertext 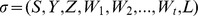.

#### De-signcrypt

Each receiver ID*_i_*, *i* = 1, 2, …, *t*, uses his/her secret key to decrypt *σ*.

1. IDi extracts his/her corresponding ciphertext information (S, Y, Z, Wi) according to his/her position in L.
2. IDi computes 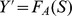 and checks whether the equation 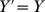 holds. If it holds, IDi continues to decrypt σ as follows; otherwise, IDi outputs ⊥.
3. IDi computes
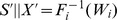
and 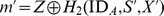.
4. Check whether the equations 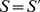 and 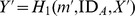 hold. If both of them hold, output 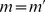; otherwise, output ⊥.

## Discussion

### 1 Correctness Analysis

#### Theorem 1

The De-signcrypt algorithm is correct.

#### Proof

Upon receiving a ciphertext *σ*, each receiver ID*_i_*, *i* = 1, 2, …, *t*, extracts his/her own corresponding ciphertext information (*S*, *Y*, *Z*, *W_i_*) from *σ*. According to the Signcrypt algorithm, we have 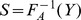, so the receiver ID*_i_* can compute 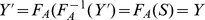. It is worth noting that only the sender, Alice, can generate the correct *Y* such that 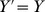 because only she knows her secret key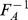. The receiver, ID*_i_*, can decrypt the ciphertext by computing 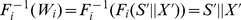 and 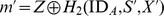. ID*_i_* can judge whether *m*′ is correct by checking whether 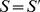 and 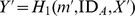 hold. Note that only ID*_i_* can obtain 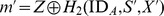
correctly because only he/she knows his/her secret key 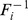. Therefore, the De-signcrypt algorithm of our scheme is correct.

### 2 Security Analysis

#### 2.1 Security of MPKC

In the last twenty years, many MPKC schemes were proposed, and they are mainly based on four basic MPKC schemes including the Matsumoto Imai (MI) cryptosystem, the Hidden Field Equation (HFE) cryptosystem, the Oil Vinegar (OV) cryptosystem and the Stepwise Triangular System [20]. Although most of them have been broken, some variants of the basic MPKC schemes, such as Rainbow and PMI+ [20], have survived known attacks like the linearization equation attack, the rank attack and the differential attack. In 2011, Hashimoto et al. [21] proposed two types of fault attacks which further weaken the security of the MPKC schemes. They detailed the fault attack on the MPKC schemes such as UOV, Rainbow, TTS and HFE, and most of the MPKC schemes were proven insecure. However, the PMI+ cryptosystem is one of the few approved cryptosystems which survived the linearization equation attack, the rank attack, the differential attack and even the fault attacks. PMI+ uses the Plus (+) method of external perturbation to prevent attacks without significantly decreasing the efficiency of the system [20]. So in our work, we used PMI+ which is based on the IP problem to construct our scheme.

Cryptosystems based on the IP problem belong to a major category of MPKC. Faugère et al. [22] gave an upper bound on the theoretical complexity of the “IP-like” problem, and presented a new algorithm to solve the IP problem when *S* and *T* are linear mappings. Bouillaguet et al. [23] proposed an improved algorithm combining the linear algebra techniques, together with Gröbner bases and statistical tools. To date, the best algorithm for the IP problem is exponential. For the IP problem used in our scheme, the attacking complexity of the best algorithm will be *Ο*(*n* ^3.5^⋅*q^n^* ^/2^) [24], where *n* is the number of variables and *q* is the cardinality of the finite field. So the IP problem will be computationally hard if we can choose the parameter properly.

With the knowledge of the most efficient attacks on the IP problem, in order to strengthen the security of our scheme, we suggest that the parameters of our scheme should satisfy the following conditions: the transformations *T* and *V* should be affine; the polynomials in *P* and *Q* should be homogeneous. In our method, for example, if we choose *n* = 16 and *q* = 2^9^, the attacking complexity should be greater than *Ο*(*n* ^3.5^⋅*q^n^* ^/2^) = 16^3.5^•(2^9^)^16/2^ = 2^86^. Usually, it is considered to be a computationally secure MPKC scheme if the attacking complexity is greater than 2^80^ [24]. Therefore, our scheme is a secure MPKC-based scheme.

Although we used PMI+ for the construction of the proposed multi-receiver signcryption scheme, there are still some other multivariate cryptosystems suitable for the construction of our scheme, such as the internally perturbed HFE cryptosystem (IPHFE) [25]. Different from PMI+, IPHFE is build by using the idea of internal perturbation. Vivien et al. [26], [27] and Ding et al. [28], [29] analyzed the security of IPHFE, and their work showed that IPHFE with appropriate parameters can withstand all known attacks. So IPHFE can be substituted for PMI+ in our construction. Due to space limitations, we do not introduce the detailed realization of the construction based on IPHFE.

#### 2.2 Message Confidentiality. Theorem 2

Confidentiality under the attack of Type 1. In the random oracle model, if an IND-CLMSC-CCA2-1 adversary *A* has a non-negligible advantage *ε* against the security of our scheme when performing 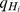
queries to random oracles *H_i_* (*i* = 1, 2), *q_ske_* Extract Secret Key queries, *q_pke_* Extract Public Key queries, *q_pkr_* Replace Public Key queries, *q_sc_* Signcrypt queries and *q_dsc_* Designcrypt queries, then there exists an algorithm *C* that can solve the MQ problem with advantage defined as:
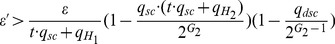
(2)where *t* is the number of receivers in the challenge set and *G*_2_ denotes the bit length of the element over *G^n^*.

#### Proof

We show how to build an algorithm *C* that solves the MQ problem with the help of an adversary *A*. Let *C* receive a random instance {*f*(*x*), *Y*_0_ =  *f*(*X*_0_)} of the MQ problem, and the goal of *C* is to compute *X*_0_. To solve this problem, *C* acts as *A*’s challenger in the IND-CLMSC-CCA2-1 game.

#### Setup

*C* sets 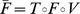 as the system public key, chooses an invertible affine transformation *T*_0_ on 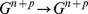, and chooses an invertible affine transformation *V*_0_ on 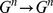 randomly. So the system partial secret key is 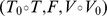, and the partial public key is 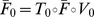. *C* sends the system parameters 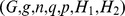, the system public key and the system partial secret key to *A*. Then *A* outputs a set of target identities, denoted by 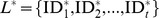. To handle *A*’s queries, *C* maintains a list *L_i_* for each *H_i_* (*i* = 1, 2) query.

#### Phase1

*C* simulates *A*’s queries as follows:

#### *H*_1_ queries

*A* can perform an *H*_1_ query on the input of (*m*, ID*_i_*, *X*, *L*) and then *C* checks the list *L*_1_. If an entry corresponding to (*m*, ID*_i_*, *X*, *L*) is present in *L*_1_, then *C* retrieves the hash value *h_i_* from *L*_1_ and returns *h_i_*. Otherwise, it returns a random number 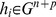 and stores the entry (*h_i_*, *m*, ID*_i_*, *X*, *L*, ∇, Δ) in *L*_1_, where the symbols ∇ and Δ denote the signature information and the encryption information of the message *m*, respectively.

#### *H*_2_ queries

*A* can perform an *H*_2_ query on the input of (ID*_s_*, *S*, *X*) for ID*_i_* and then *C* checks the list *L*_2_. If an entry corresponding to ID*_i_* is present in *L*_2_, then *C* retrieves *Z_i_* from *L*_2_ and returns *Z_i_*. Otherwise, it returns a random number *Z_i_* and stores the entry (*Z_i_*, ID*_s_*, *S*, *X*, ID*_i_*, Λ, □, ◊, *b_i_* = 0) in *L*_2_, where the symbols Λ, □ and ◊ denote the public key *F_i_*, the secret parameters *T_i_* and *V_i_*, respectively. The bit *b_i_* is a flag bit used to denote whether the public keys have been replaced or not.

#### Extract Secret Key queries

*A* can perform an Extract Secret Key query on the input of ID*_i_*. *C* first checks whether 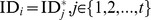 holds. If 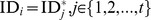
holds, then *C* aborts the query. Otherwise, *C* retrieves the entry (*Z_i_*, ID*_s_*, *S*, *X*, ID*_i_*, *F_i_*, *T_i_*, *V_i_*, *b_i_* = 0) from *L*_2_. If *b_i_* = 0, then *C* returns the secret key 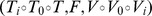; otherwise, the public key of the identity ID*_i_* has been replaced and in this case, *C* asks *A* for the new secret parameters (*T_i_*, *V_i_*), computes the new secret key 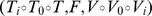 and returns it to *A*.

#### Extract Public Key queries

*A* can perform an Extract Public Key query on the input of ID*_i_* and then *C* checks *L*_2_. If an entry corresponding to ID*_i_* is present in *L*_2_, then *C* retrieves *F_i_* from *L*_2_ and returns *F_i_*. Otherwise, *C* chooses 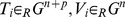, returns the public key 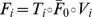 and updates the entry corresponding to ID*_i_* in *L*_2_ with *T_i_*, *V_i_* and *F_i_*.

#### Replace Public Key queries

When *A* performs a Replace Public Key query on the input of 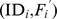, *C* searches the corresponding entry 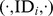 in *L*_2_. If the entry is found, then *C* replaces the public key in the entry corresponding to ID*_i_* in *L*_2_ with 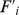 and sets the flag bit *b_i_* to 1. Otherwise, *C* generates the public key using the Extract Public Key query and then replaces the public key of ID*_i_* with 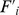.

#### Signcrypt queries

*A* can perform a Signcrypt query on the input of (*m*, ID*_s_*, *L* = {ID*_R_*_1_, ID*_R_*_2_, …, ID*_Rt_*}). If ID*_s_* =  ID*_Ri_*, 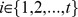, or if 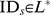 and at least one 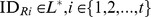, then *C* aborts the query. Otherwise, *C* knows the secret key of the sender and performs the computations as the signcryption algorithm to return the ciphertext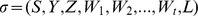. If 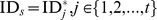, *C* does not know the secret key of the sender and in this case, it generates the ciphertext as follows:

First, *C* retrieves the entry (*⋅*, ID*_s_*, *F_s_*, *T_s_*, *V_s_*, *b_s_*) from *L*_2_ and chooses 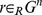 and 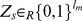. *C* computes 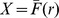, extracts *Y* = *h_s_* by calling the oracle *H*_1_ with the input 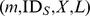
and computes the signature *S*. Then, *C* retrieves the corresponding entry 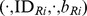 in *L*_2_ and then computes *W_i_* = *F_i_*(*S*||*X*) and *Z* = *Z_s_*⊕*m*. *C* updates the corresponding entry in *L*_1_. Note that if *b_i_* = 1, then the public key of the receiver has been replaced and in this case, the challenger asks *A* for (*T_i_*, *V_i_*) and uses it in place of the old value stored in the entry. Finally, add 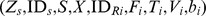 in *L*_2_ (*C* fails if *H*_2_ is already defined on any of such entries, but this happens only with probability 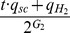). At last, *C* sends 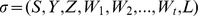 to *A*.

#### Designcrypt queries

When *A* submits a ciphertext 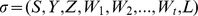, a receiver’s identity ID*_R_* and a sender’s identity ID*_s_*, *C* extracts (*S*, *Y*, *Z*, *W_i_*, *L*) from *σ*. If 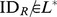, then *C* knows the secret key of ID*_R_* and hence designcrypts *σ* using the De-signcrypt Algorithm. Otherwise, *C* searches all entries (*Y*, ⋅, ID*_s_*, ⋅, *L*, *⋅*, *Z*) in *L*_1_, and if no such entries exist, the symbol ⊥ is returned to indicate that the ciphertext is invalid. Meanwhile, *C* searches the entry (*Z_i_*, ID*_s_*, *S*, *X*, ID*_s_*, *F_s_*, *T_s_*, *V_s_*, *b_s_*) in *L*_2_, and if it is not found, *C* rejects the ciphertext *σ*. If the ciphertext *σ* passes the above verification, *C* computes 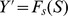,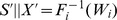, and 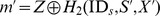. If 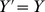
and
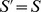
hold, and 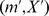 passes the verification, then *C* returns *m*; otherwise, *C* rejects *σ*. Note that a valid ciphertext is rejected with probability at most 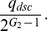


#### Challenge

*A* outputs two messages *m*_0_ and *m*_1_ together with an arbitrary sender’s identity 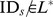 on which *A* wishes to be challenged. *C* selects a bit 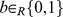 and sends *m_b_* to the *t* target identities denoted by 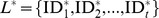. *C* chooses 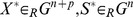
and 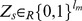, sets 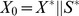 and then computes 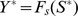,
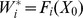
and 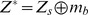. Then, *C* responds with the ciphertext 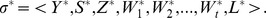


#### Phase2

*A* performs new queries as in Phase 1. However, *A* is not allowed to ask Designcrypt queries on *σ* ^*^ for 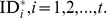


#### Guess

At the end of the game, *A* returns his/her guess result. *C* ignores the answer to *A*’s guess. According to the above discussion, we know that as long as the simulation of the attacker’s environment is perfect, the probability that *A* asks the value of *W_i_* = *F_i_*(X^*^||S^*^), *i* = 1, 2, …, *t*, by the *H*_1_ oracle is the same as the probability in a real attack. *C* fetches a random entry (*h_i_*, *m*, ID*_i_*, *X*, *L*, *S*, *W_i_*) from *L*_1_. With probability 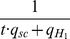
(as *L*_1_ contains no more than *t*⋅*q_sc_*+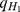 elements by our construction), the chosen entry contains the right element 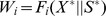. *C* returns *X*_0_ as a solution to the MQ problem.

Now, we analyze the probability of *C*’s success. Let *E* be the event that *A* outputs the correct bit b^*^ = *b*.

Simulation fails if any of the following events occurs:

*E*_1_: Extract Secret Key query is executed for some chosen challenge identity.

*E*_2_: Both the sender and at least one of receivers belong to the challenge set in some Signcrypt query.

*E*_3_: The *H*_2_ oracle collides in Signcrypt queries.

*E*_4_: *C* rejects a valid ciphertext in some Designcrypt query.

According to the above discussion, we know that *Pr*[*E*] = *ε*, where *E* implies that *E*_1_ *and E*_2_ never occur, that is, 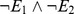. Also, we have 
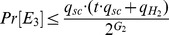
 since *A* conducts a total of *q_sc_* Signcrypt queries and there are at most *t*⋅*q_sc_*+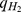 entries in *L*_2_. 
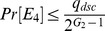
 represents the probability of rejection of valid ciphertexts.

The event *E*_5_ implies that *C* chooses the correct entry from *L*_1_ in the Guess Phase. And we know that 
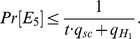
 So, the advantage 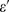 of *C* is defined as:
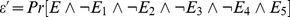
(3)


Therefore, we obtain
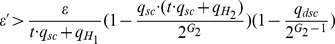
(4)


#### Theorem 3

Confidentiality under the attack of Type 2. In the random oracle model, if an IND-CLMSC-CCA2-2 adversary *A* has a non-negligible advantage *ε* against the security of our scheme when performing 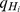
queries to random oracles *H_i_* (*i* = 1, 2), *q_ske_* Extract Secret Key queries, *q_pke_* Extract Public Key queries, *q_sc_* Signcrypt queries and *q_dsc_* Designcrypt queries, then there exists an algorithm *C* that can solve the MQ problem with an advantage 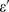 defined as:
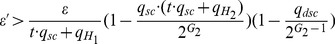
(5)where *t* is the number of receivers in the challenge set and *G*_2_ denotes the bit length of the element over *G^n^*.

The attacker has access to the master key, but cannot perform public key replacement under the attack of Type 2. The proof is similar to that of Theorem 2.

#### 2.3 Unforgeability. Theorem 4

Unforgeability under the attack of Type 1. In the random oracle model, if an SUF-CLMSC-CMA-1 adversary *A* has a non-negligible advantage *ε* against the security of our scheme when performing 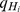
queries to random oracles *H_i_* (*i* = 1, 2), *q_ske_* Extract Secret Key queries, *q_pke_* Extract Public Key queries, *q_pkr_* Replace Public Key queries, *q_sc_* Signcrypt queries and *q_ver_* Verify queries, then there exists an algorithm *C* that can solve the IP problem with an advantage 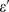 defined as:
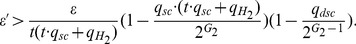
(6)where *t* is the number of receivers in the challenge set and *G*_2_ denotes the bit length of the element over *G^n^*.

#### Proof

We show how to build an algorithm *C* that solves the IP problem with the help of an adversary *A*. Let *C* receive a random instance 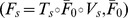 of the IP problem, and the goal of *C* is to compute (*T_s_*, *V_s_*). To solve this problem, *C* acts as *A*’s challenger in the SUF-CLMSC-CMA-1 game.

#### Setup

*C* sets 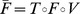 as the system public key, and chooses an invertible affine transformation *T*_0_ on 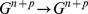 and an invertible affine transformation *V*_0_ on 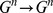 randomly. So the system partial secret key is 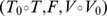, and the partial public key is 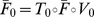. *C* sends the system parameters 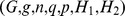, the system public key and the system partial secret key to *A*. Then *A* outputs a set of target identities, denoted by 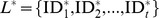. To handle *A*’s queries, *C* maintains a list *L_i_* for each *H_i_* (*i* = 1, 2) query.

#### Attack

*C* simulates *A*’s queries as follows:

#### *H*_1_ queries

*A* can perform an *H*_1_ query on the input of (*m*, ID*_i_*, *X*, *L*) and then *C* checks the list *L*_1_. If an entry corresponding to (*m*, ID*_i_*, *X*, *L*) is present in *L*_1_, then *C* retrieves *h_i_* from *L*_1_ and returns *h_i_*. Otherwise, it returns a random number 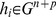 and stores the entry (*h_i_*, *m*, ID*_i_*, *X*, *L*, ∇, Δ) in *L*_1_, where the symbols ∇ and Δ denote the signature information and the encryption information for message *m*, respectively.

#### *H*_2_ queries

*A* can perform an *H*_2_ query on the input of (ID*_s_*, *S*, *X*) for ID*_i_* and then *C* checks the *L*_2_. If an entry corresponding to ID*_i_* is present in *L*_2_, then *C* retrieves *Z_i_* from *L*_2_ and returns *Z_i_*. Otherwise, it returns a random number *Z_i_* and stores the entry (*Z_i_*, ID*_s_*, *S*, *X*, ID*_i_*, Λ, □, ◊, *b_i_* = 0) in *L*_2_, where the symbols Λ, □ and ◊ denote the public key *F_i_*, the secret parameters *T_i_* and *V_i_*, respectively. The bit *b_i_* is a flag bit used to denote whether the public keys have been replaced or not.

#### Extract Secret Key queries

*A* can perform an Extract Secret Key query on the input of ID*_i_*, and *C* first checks whether 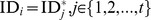 holds. If 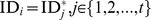
holds, then *C* aborts the query. Otherwise, *C* retrieves the entry (*Z_i_*, ID*_s_*, *S*, *X*, ID*_i_*, *F_i_*, *T_i_*, *V_i_*, *b_i_* = 0) from *L*_2_. If *b_i_* = 0, then *C* returns the secret key 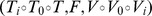. Otherwise, the public key of the identity ID*_i_* has been replaced and in this case, *C* asks *A* for the new secret parameters (*T_i_*, *V_i_*), computes the new secret key 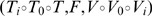 and returns it to *A*.

#### Extract Public Key queries

*A* can perform an Extract Public Key query on the input of ID*_i_* and then *C* checks *L*_2_. If an entry corresponding to ID*_i_* is present in *L*_2_, then *C* retrieves *F_i_* from *L*_2_ and returns *F_i_*. Otherwise *C* chooses *T_i_*∈*_R_ G^n^* ^+*p*^ and *V_i_*∈*_R_ G^n^*, returns the public key 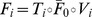 and updates the entry corresponding to ID*_i_* in *L*_2_ with *T_i_*, *V_i_* and *F_i_*.

#### Replace Public Key queries

When *A* performs a Replace Public Key query on the input of 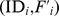, *C* searches the corresponding entry 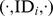 in *L*_2_. If the entry is found, then *C* replaces the public keys in the entry corresponding to ID*_i_* in *L*_2_ with 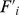 and sets the flag bit *b_i_* to 1. Otherwise, *C* generates the public key using Extract Public Key query and then replaces the public key of ID*_i_* with 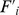.

#### Signcrypt queries

*A* can performs a Signcrypt query on the input of (*m*, ID*_s_*, *L* = {ID*_R_*_1_, ID*_R_*_2_, …, ID*_Rt_*}). If ID*_s_* = ID*_Ri_*,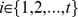, or if 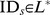 and at least one 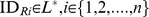, then *C* aborts the query. If 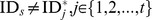, then *C* knows the secret key of the sender and performs the computations as the Signcrypt algorithm to return the ciphertext 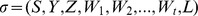. If 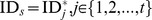, *C* does not know the secret key of the sender and hence it generates the ciphertext as follows:

First, *C* retrieves the entry (*⋅*, ID*_s_*, *F_s_*, *T_s_*, *V_s_*, *b_s_*) from *L*_2_ and chooses 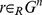
and 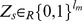. *C* computes 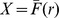, extracts *Y* = *h_s_* by calling the oracle *H*_1_ with the input (*m*, ID*_s_*, *X*, *L*) and computes the signature *S*. Then, *C* retrieves the corresponding entry 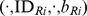 in *L*_2_ and then computes *W_i_* = *F_i_*(*S*||*X*) and *Z* = *Z_s_*⊕*m*. *C* updates the corresponding entry in *L*_1_. Note that if *b_i_* = 1, then the public key of the receiver has been replaced and in this case, the challenger asks *A* for (*T_i_*, *V_i_*) and uses it in place of the old value stored in the entry. Finally, add 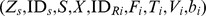 in *L*_2_ (*C* fails if *H*_2_ is already defined on any of such entries, but this happens only with probability 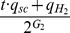). At last, *C* sends 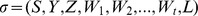 to *A*.

#### Verify queries

When *A* submits a ciphertext 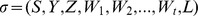, a receiver’s identity ID*_R_* and a sender’s identity ID*_s_*, *C* extracts (*S*, *Y*, *Z*, *W_i_*, *L*) from *σ*. If 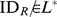, then *C* knows the secret key of ID*_R_* and hence designcrypts *σ* using the De-signcrypt Algorithm. Otherwise, *C* searches all entries (*Y*, ⋅, ID*_s_*, ⋅, *L*, *⋅*, *Z*) in *L*_1_, and if no such entries exist, the symbol ⊥ is returned to indicate that the ciphertext is invalid. Meanwhile, *C* searches the entry (*Z_i_*, ID*_s_*, *S*, *X*, ID*_s_*, *F_s_*, *T_s_*, *V_s_*, *b_s_*) in *L*_2_, and if it is not found, *C* rejects the ciphertext *σ*. If the ciphertext *σ* passes the above verification, *C* computes 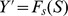,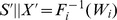 and 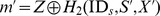. If 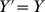
and 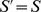
hold, and 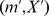 passes the verification test, then *C* accepts *σ*; otherwise, *C* rejects *σ*. Note that a valid ciphertext is rejected with probability at most 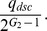


#### Forge

After a polynomial-bounded number of queries, the adversary *A* outputs forged ciphertext 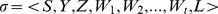 (a receiver list *L* = {ID*_R_*_1_, ID*_R_*_2_, …, ID*_Rt_*}, and at least one 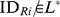) and the sender’s identity 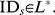


According to the above discussion, we know that as long as the simulation of the attacker’s environment is perfect, the probability that *A* asks the value of (*T_s_*, *V_s_*) by the *H*_2_ oracle is the same as the probability in a real attack. *C* fetches a random entry (*Z_i_*, ID*_s_*, *S*, *X*, ID*_i_*, *F_i_*, *T_i_*, *V_i_*, *b_i_*) from *L*_2_. With probability 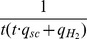 (as *L*_2_ contains no more than *t*⋅*q_sc_*+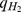
elements by our construction, and *C* chooses ID*_s_* with probability 1/*t*), the chosen entry contains the right element (*T_s_*, *V_s_*). *C* returns (*T_s_*, *V_s_*) as the solution to the IP problem.

Now, we analyze the probability of *C*’s success. Let *E* be the event that the forged ciphertext passes verifications.

Simulation fails if any of the following events occurs:

*E*_1_: Extract Secret Key query is executed for some chosen challenge identity.

*E*_2_: Both sender and at least one of receivers belong to the challenge set in some Signcrypt query.

*E*_3_: The *H*_2_ oracle collides in Signcrypt queries.

*E*_4_: *C* rejects a valid ciphertext in Verify queries.

According to the above discussion, we know that *Pr*[*E*] = *ε*, where *E* implies that *E*_1_ and *E*_2_ never occur, that is, 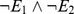. Also, we have 
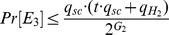
, since *A* conducts a total of *q_sc_* Signcrypt queries and there are at most *t*⋅*q_sc_*+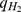 entries in *L*_2_. 
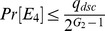
 represents the probability of rejection of valid ciphertexts.

The event *E*_5_ implies that *C* chooses the correct entry from *L*_2_ in the last Verify Phase. And we know that 
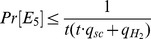
. So, the advantage 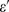 of *C* is defined as:
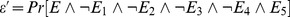
(7)


Therefore, we obtain
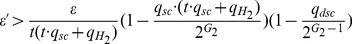
(8)


#### Theorem 5

Unforgeability under the attack of Type 2. In the random oracle model, if an SUF-CLMSC-CMA-2 adversary *A* has a non-negligible advantage *ε* against the security of our scheme when performing 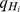
queries to random oracles *H_i_* (*i* = 1, 2), *q_ske_* Extract Secret Key queries, *q_pke_* Extract Public Key queries, *q_sc_* Signcrypt queries and *q_ver_* Verify queries, then there exists an algorithm *C* that can solve the IP problem with an advantage 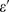 defined as:
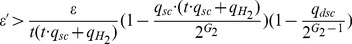
(9)where *t* is the number of receivers in the challenge set and *G*_2_ denotes the bit length of the element over *G^n^*.

The attacker has access to the master key, but cannot perform public key replacement under the attack of Type 2. The proof is similar to that of Theorem 4.

#### 2.4 Backward Secrecy

Each time Alice sends a message *m* to receivers, she chooses 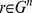 randomly as the session key. Even though she sends the same message *m*, the corresponding ciphertext *σ* will be different in different sessions. So the new receiver who joins the group later does not have the previous value 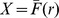 which is computed for the message *m*, and thus he/she can not obtain the previous message *m*. Therefore, our scheme is backward secure.

#### 2.5 Forward Secrecy

Forward secrecy means that the members who have quitted the group are not able to know the later session keys. In our scheme, the session key *r* is randomly chosen in each session. When some member of the group quits the group, the sender will compute the partial key for the rest members again, which guarantees that the members who have quitted the group cannot obtain the plaintext message from the later ciphertext. So our scheme is forward secure.

#### 2.6 Non-repudiation

According to Theorem 4 and Theorem 5, our scheme is unforgeable. Suppose that Alice signcrypts a message *m*. If others want to repudiate her signature *S*, they have to solve the MQ problem to get the secret key of Alice, and it is computationally infeasible because the MQ problem is an NP-hard problem. Therefore, only Alice knows her secret key and others can not repudiate her behavior of signcrypting the message *m*. So our scheme is non-repudiation.

#### 2.7 Public Verifiability

The proposed scheme provides public verifiability of ciphertext source, which is an important requirement in broadcast communications. Any third party can be convinced of the sender of the ciphertext *σ* by recovering 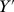 in the second step of the de-signcryption phase and checking whether the equation 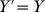
holds. This is in fact due to the unforgeability of the signature. This verification procedure does not involve the knowledge of messages or the receiver’s secret key but only the ciphertext *σ*. Hence, our scheme supports public verifiability.

### 3 Performance Comparison

In this section, we shall compare our scheme with the existing schemes [8]–[10], [12] in performance. We mainly consider the computation and communication cost.

The proposed scheme does not involve any bilinear pairing operations, exponentiation operations and multiplications in groups. In the signcryption phase, it needs only two hash operations, (*t*+2) MQ-mapping (it means the mapping operation on the multivariate quadratic equations) operations and one XOR operation, while in the de-signcryption phase, it needs two hash operations, two MQ-mapping operations and one XOR operation. The MQ-mapping operations are linear operations and have much lower computation complexity than bilinear pairing operations and exponentiation operations. According to the above analysis, the computation complexity of our scheme is *O*(*t*+4). The ciphertext of our scheme is (*t*+1)*G*_1_+(*t*+1)*G*_2_+|*m*| bits in length, where *t* is the number of receivers, *G*_2_ is the bit length of the element over *G^n^*, and *G*_1_ is the bit length of the element over *G^n^* ^+*p*^. Compared with the representative CLMSC scheme [12], the new scheme has lower computation complexity without bilinear pairing operation needed. We also compare our scheme with the naive extension of schemes [8]–[10] for multi-receiver setting in Table 1, in which par denotes pairing operation, exp denotes exponentiation operation and ciphertext-size denotes the bit length of the ciphertext. The comparisons are summarized in Table 1.

**Table 1:** Comparison of our scheme and the existing ones.

scheme	MQ-mapping	par	exp	hash	ciphertext-size
Li et al.’s [8]	0	2	*t+*1	2*t*+2	2*t*|*I*|+*t*|*m*|
Selvi et al.’s [9]	0	0	5*t*+7	3t+3	2*t*|*Z_q_*|+2|*I*|+ *t*|*m*|
Jing et al.’s [10]	0	0	3*t*+2	2*t*+2	2*t*|*I*|+*t*|*m*|
Selvi et al.’s [12]	0	2	2*t*+2	*t*+7	(2*t*+1)|*Z_q_*|+|*I*|
Ours	*t*+4	0	0	4	(*t*+1)*G*_1_+(*t*+1)*G*_2_+|*m*|

*t* denotes the number of receivers, |*Z_q_*| denotes the bit length of elements in finite field *Z_q_*, |*I*| denotes the bit length of elements in group *I*,

|*m*| denotes the bit length of message *m*, *G*_1_ denotes the bit length of elements in *G^n^* ^+*p*^, *G*_2_ denotes the bit length of elements in *G^n^.*

According to the above analyses, the proposed scheme is more efficient than the existing ones, and it is also provably secure in the random oracle model. The proposed scheme is a very useful tool in multicast communication. With the rapid development of wireless networks, it is particularly important to transfer instruction data from the control center to multiple intelligent terminals securely [30]. The control center needs to encrypt the sensitive information to prevent it from being eavesdropped and cracked before sending it to intelligent terminals, while intelligent terminals need to judge whether the received instruction is from the trusted entity. To solve this security problem, we must take both the security requirements and the performance of the intelligent terminals into account, because intelligent terminals are generally characterized by low power consumption, low computing power and narrow communication bandwidth, which make the traditional identity-based scheme not suitable for them. Through the analyses about the security and performance of our scheme, it can be concluded that our scheme can better address these issues and it is in line with the characteristics of intelligent terminals.

### Conclusions

As one of the alternative cryptosystems, multivariate public key cryptography can resist quantum attack, and has been researched by scholars extensively. In this paper, we employ multivariate public key cryptography to propose a new construction of the certificateless multi-receiver signcryption scheme, called a quantum attack-resistent certificateless multi-receiver signcryption scheme. The new scheme inherits the security of multi-variable cryptosystems that could resist quantum attack, and it avoids the certificate management and the key escrow problem. We proved its security under the hardness of the MQ problem and its unforgeability under the IP assumption in the random oracle model. In addition, the scheme also has security properties such as forward secrecy, backward secrecy, non-repudiation and public verifiability. Analyses show that the proposed scheme is more efficient than the existing ones. Although our scheme is constructed by using PMI+, there are still some other multivariate cryptosystems like IPHFE suitable for our construction. In the future work, we will construct the multi-receiver signcryption scheme by using IPHFE or other better multivariate cryptosystem, and compare the performance of the new scheme with that of the scheme proposed in this paper.
